# Clinicopathologic analysis of lymph node status in superficial esophageal squamous carcinoma

**DOI:** 10.1186/s12957-016-1016-0

**Published:** 2016-10-12

**Authors:** Yue Zhou, Junjie Du, Hai Li, Jinhua Luo, Liang Chen, Wei Wang

**Affiliations:** 1 Department of Cardiothoracic Surgery, First Affiliated Hospital of Nanjing Medical University, No. 300 Guangzhou Road, Nanjing, 210029 China; 2 Department of Pathology, First Affiliated Hospital of Nanjing Medical University, No. 300 Guangzhou Road, Nanjing, 210029 China

**Keywords:** Superficial esophageal squamous carcinoma, Lymph node metastasis, Prognostic

## Abstract

**Background:**

Endoscopic approaches are gradually considered as a reliable treatment of intramucosal esophageal squamous carcinoma. However, endoscopic resection (ER) is limited by the potential lymph node metastasis (LNM) at various depths of mucosal and submucosal invasion.

**Methods:**

We conducted a retrospective review of 498 patients with pT1 superficial esophageal squamous carcinoma (SESC) who underwent surgical resection from January 2008 to August 2015. Pathological characteristics of tumors including location, size, appearance, differentiation, invasion depth, and nodal status were reviewed, and risk factors were analyzed.

**Results:**

LNM was found in 0.0, 2.7, 6.3, 18.2, 15.9, and 34.3 % of the m1, m2, m3, sm1, sm2, and sm3 lesions, respectively. Univariate logistic regression identified the presence of the tumor size > 2 cm (*p* < 0.05), the presence of the poor tumor differentiation (*p* < 0.05), and the depth of tumor invasion (*p* < 0.05) and angiolymphatic invasion (*p* < 0.05) to be the important risk factors associated with the prevalence of tumor-positive lymph nodes. These findings were confirmed in multivariate logistic regression as independent predictors for LNM.

**Conclusions:**

ER is considered as a reliable treatment of m1 to m2 lesions. Radical surgical resection (SR) is the standard and irreplaceable therapy of sm1 to sm3 lesions. Patients with m3 lesions should undergo ER as the initial procedure for diagnosis. And this treatment is supported only by a successful description of the tumor’s characteristics, including (1) only muscularis mucosa invasion and without invasion of the resection margins and (2) without any risk predictors for LNM. Otherwise, SR is recommended.

## Background

With the recent progress in endoscopic techniques, the incidence of superficial esophageal squamous carcinoma (SESC) is increasing. Patients with SESC may expect a more favorable prognosis compared with those who have advanced esophageal squamous carcinoma. The tumor depth of these SESC is subdivided into six successive layers (m1: intraepithelial tumors; m2: tumors invading the lamina propria; m3: tumors invading the muscularis mucosa; sm1: tumors invading the most superficial one third of the submucosa; sm2: tumors invading the middle one third of the submucosa; sm3: tumors invading deeper than the sm2 level) [1]. Due to the low prevalence of lymph node metastasis (LNM) of m1 and m2, endoscopic resection (ER) is gradually considered a reliable treatment [2–5] with acceptable low morbidity and mortality [6]. These excellent outcomes have promoted some authors to consider that m3 and sm1 tumors would be also feasible. On the other hand, others argued that radical surgical resection (SR; the radical esophagectomy with lymphadenectomy) is the standard therapy for SESC considering the potential LNM [7, 8], although its high risk of morbidity and mortality remain debatable [5, 6, 9–13], not to mention the negative impact on long-term quality of life.

Therefore, the accurate prediction of lymph node involvement in different subsets of SESC would give the patient a favorable opportunity to receive more reasonable treatment. The aim of this study was (1) to clarify the relationship between clinicopathological risk factors and LNM and (2) to identify the best candidate patients for ER treatment.

## Methods

### Study patients

Consecutive patients with esophageal squamous carcinoma who underwent surgical resection at our hospital between January 2008 and August 2015 were reviewed retrospectively. A total of 498 patients with pT1 SESC who underwent radical esophagectomy with lymphadenectomy were enrolled for this study. All patients were examined with upper gastrointestinal endoscopy/endoscopic ultrasound with biopsy, standard chest radiograph, and multi-slice computed tomography (MSCT) of the chest and upper abdomen to exclude the presence of metastatic disease, as well as an assessment of cardiopulmonary reserve to exclude those patients with surgical contraindication. No patients received preoperative neoadjuvant.

### Pathological evaluation

Pathologic examination of the resected specimens was performed at the Department of Pathology of our institution. The standard procedure for processing esophagectomy specimens includes immediate assessment of the specimen to measure tumor dimensions followed by formalin fixation overnight. For T1 tumors with a mass that is seen grossly, the entire mass is submitted for histologic examination. Each resected specimen was cut into 15 × 2 mm blocks, fixed in formalin, embedded in paraffin, and cut into 5-μm slices. Sections were routinely stained with hematoxylin and eosin. For those angiolymphatic invasions that were considered, immunohistochemical analysis with CD31, CD34, and D2-40 was performed to seek evidence of angiolymphatic infiltrations (CD31- and CD34-positive) and lymphatic invasions (further D2-40-positive). The description of the tumor which included tumor location, tumor size, pathologic appearance, tumor differentiation, tumor depth, and nodal status was pathologically evaluated. Additionally, the subdivided depth of tumor invasion was classified into six levels: m1 to m3 and sm1 to sm3, as described above [1]. Tumor differentiation was classified into five groups including I (well differentiated), I–II, II (moderately differentiated), II–III, and III (poorly differentiated). Tumor differentiation and/or growth pattern may be categorized as absent variables in those patients with very early stage tumor like high-grade dysplasia/carcinoma in situ. Other variables including multicentric invasive lesions and angiolymphatic invasion were also examined in order to find out the relationship with LNM. Tumors classified as undifferentiated (IV), basaloid squamous cell carcinoma, glanular cell, or spindle cell were excluded from this study. The pathologic tumor stage was presented according to the 7th edition of the American Joint Committee on Cancer TNM classification [14].

### Statistical analysis

All enrolled patients were divided into two groups according to their results of LNM (group I with negative LNM and group II with positive LNM). Variables subjected to statistical analysis were evaluated, including age, gender, tumor location, tumor size, pathologic appearance, tumor differentiation, tumor depth (m1 to m3 and sm1 to sm3), multicentric invasion, and angiolymphatic invasion. A *χ* ^2^ test was used to compare categorical variables. The Student *t* test and analysis of variance were used for comparison of continuous variables. Univariate logistic regression and multivariate logistic regression analysis were used to identify the risk factors and independent predictors of LNM, respectively. All *p* values were two-sided, and the significance level was set at *p* < 0.05. Continuous data were presented as mean ± SD. All calculations were performed using the STATA 10.0 software (StataCorp, College Station, TX, USA).

## Results

### Patient demographics and clinicopathologic characteristics

A total of 498 patients with pT1 SESC were enrolled for this study. Table 1 shows the patients’ demographics and clinical characteristics. Mean patient age was 60.74 years (range from 36 to 80). The tumor originated at the cervical esophagus in 16 patients, upper thoracic esophagus in 90, middle thoracic esophagus in 248, and lower thoracic esophagus in 144. Tumor with erosion type was found in 99 patients, ulcerative type in 123, fungating type in 84, other types in 66, and with unavailable pathologic appearance in 126 patients. The tumor differentiation included I (24 patients), I–II (71 patients), II (185 patients), II–III (86 patients), III (46 patients), and unavailable (86 patients). Eleven patients (2.2 %) were found with tumor multicentric invasion. And there were 31 patients (6.2 %) with tumor angiolymphatic invasion. All patients were proved to be SESC pathologically. Among them, as shown in Table 2, 411 patients with negative LNM were in group I and 87 with positive LNM in group II. The characteristics in these two groups were compared in terms of age, gender, tumor location, tumor size, pathologic appearance, tumor differentiation, tumor depth (m1 to sm3), multicentric invasion, and angiolymphatic invasion. Group II had a significantly larger tumor size than that in group I (2.31 ± 1.09 vs 1.73 ± 0.97, *p* < 0.05). There were remarkable statistic differences (*χ* ^2^ test, *p* < 0.05) in tumor differentiation (I, I–II, II, II–III, III) and in angiolymphatic invasion (absent, present) between two groups. The lymph node involvement in group II increased significantly (*p* < 0.05) from m1 to sm3 (Table 2).

**Table 1:** Patient demographics and clinical characteristics

Variables	Value (%)
Number	498
Age (years) mean ± SD (range)	60.74 ± 7.21 (36–80)
Gender (M/F)	367/131
Tumor location (%)	
Cervical	16 (3.2)
Upper thoracic	90 (18.1)
Middle thoracic	248 (49.8)
Lower thoracic	144 (28.9)
Tumor size (cm)	
Mean ± SD (range)	1.83 ± 1.02 (0.1–6.5)
Pathologic appearance (%)	
Erosive type	99 (19.9)
Ulcerative type	123 (24.7)
Fungating type	84 (16.9)
Other types	66 (13.3)
Unavailable	126 (25.3)
Differentiation (%)	
I	24 (4.8)
I–II	71 (14.3)
II	185 (37.1)
II–III	86 (17.3)
III	46 (9.2)
Unavailable	86 (17.3)
Depth of invasion (%)	
m1	43 (8.6)
m2	37 (7.4)
m3	80 (16.1)
sm1	88 (17.7)
sm2	113 (22.7)
sm3	137 (27.5)
Multicentric invasion (%)	
Absent	487 (97.8)
Present	11 (2.2)
Angiolymphatic invasion (%)	
Absent	467 (93.8)
Present	31 (6.2)

*SD* standard deviation

**Table 2:** Demographics of patients in the negative LNM and positive LNM groups

Variables	Group		*p* value
	Negative LNM (group I)	Positive LNM (group II)	
Number	411	87	
Age (years)			
Mean ± SD	60.91 ± 7.19	59.91 ± 7.26	0.237
Gender			
Male	301	66	0.613
Female	110	21	
Tumor location			
Cervical	15	1	0.147
Upper thoracic	71	19	
Middle thoracic	212	36	
Lower thoracic	113	31	
Tumor size (cm)			
Mean ± SD	1.73 ± 0.97	2.31 ± 1.09	<0.05*
Pathologic appearance^a^			
Erosive type	85	14	0.175
Ulcerative type	98	25	
Fungating type	61	23	
Other types	53	13	
Differentiation^b^			
I	22	2	<0.05*
I–II	67	4	
II	154	31	
II–III	52	34	
III	33	13	
Depth of invasion			
m1	43	0	<0.05* m1 to m2 vs m3 to sm3: *p* < 0.05 m1 to m3 vs sm1 to sm3: *p* < 0.05 m1 to m2 vs m3 to sm1 vs sm2 to sm3: *p* < 0.05
m2	36	1	
m3	75	5	
sm1	72	16	
sm2	95	18	
sm3	90	47	
Multicentric invasion			
Absent	404	83	0.095
Present	7	4	
Angiolymphatic invasion			
Absent	396	71	<0.05*
Present	15	16	

*SD* standard deviation

*Statistically significant

^a^Data available in 372 patients

^b^Data available in 412 patients

### Pathological features based on invasion depth

LNM was found in none of the 43 patients with m1 lesions, 1 of the 37 patients (2.7 %) with m2 lesions, 5 of the 80 patients (6.3 %) with m3 lesions, 16 of the 88 patients (18.2 %) with sm1 lesions, 18 of the 113 patients (15.9 %) with sm2 lesions, and 47 of the 137 patients (34.3 %) with sm3 lesions. Lymph node involvement increased significantly (*p* < 0.05) from m1 to sm3, including m1 to m2 vs m3 to sm3 (*p* < 0.05), m1 to m3 vs sm1 to sm3 (*p* < 0.05), and m1 to m2 vs m3 to sm1 vs sm2 to sm3 (*p* < 0.05) (Table 2). Table 3 summarizes the number and region of involved lymph nodes in relation to the invasion depth in SESC. We divided the region of LNM into four parts including the cervical region, upper thoracic region, middle and lower thoracic region, and abdominal region. In cases with LNM, we observed that the number and region of involved nodes increased in correlation with the depth of invasion.

**Table 3:** Number and region of involved lymph nodes in relation to the cancerous depth in SESC of the thoracic esophagus treated by thoracotomy

	m1	m2	m3	sm1	sm2	sm3
Total number	43	37	80	88	113	137
Number of positive LN (%)						
≤2	0	1 (2.7)	4 (5)	14 (15.91)	15 (13.27)	29 (21.17)
3–6	0	0	1 (1.25)	2 (2.27)	3 (2.65)	16 (11.68)
≥7	0	0	0	0	0	2 (1.46)
Number of patients regarding regions of positive LN (%)						
One region	0	1 (2.7)	4 (5)	15 (17.05)	12 (10.62)	32 (23.36)
Two regions	0	0	1 (1.25)	1 (1.14)	6 (5.31)	15 (10.95)
Three regions	0	0	0	0	0	0

### Relationships between pathological findings and LNM

Univariate analysis showed that the tumor size was more than 2 cm (*p* < 0.05); the poor tumor differentiation (I + I–II vs II + II–III + III, *p* < 0.05), the depth of tumor invasion (m1 to m3 vs sm1 to sm3, *p* < 0.05), and angiolymphatic invasion (*p* < 0.05) were the important risk factors associated with the prevalence of tumor-positive lymph nodes (Table 4). And these results were also found in multivariate analysis which meant the tumor size was more than 2 cm (*p* = 0.003); the poor tumor differentiation (I + I–II vs II + II–III + III, *p* = 0.004), the depth of tumor invasion (m1 to m3 vs sm1 to sm3, *p* = 0.005), and angiolymphatic invasion (absent vs present, *p* < 0.05) were four independent predictors for LNM (Table 5). But other pathological characteristics, including age (*p* = 0.494), gender (*p* = 0.613), tumor location (*p* = 0.669), and multicentric invasion (*p* = 0.109), were not associated with LNM as indicated by the results of univariate logistic regression analysis which is not mentioned in Table 5. In brief, patients presenting as LNM-positive have larger, more poorly differentiated, and deeper invaded tumors with higher angiolymphatic invasion.

**Table 4:** Univariate analysis of the risk factors for lymph node metastases

Variables	OR (95 % CI)	*p* value
Gender		
Male vs female	0.87 (0.51–1.49)	0.613
Age		
≤60 vs >60	0.85 (0.54–1.35)	0.494
Tumor location		
Cervical/upper vs middle/lower	0.89 (0.51–1.54)	0.669
Tumor size		
≤2 vs >2 cm	2.56 (1.59–4.13)	<0.05*
Differentiation		
I + I–II vs II + II–III + III	2.85 (1.73–4.71)	<0.05*
Depth of invasion		
m1 to m3 vs sm1 to sm3	8.09 (3.45–18.98)	<0.05*
Multicentric invasion		
Absent vs present	2.78 (0.80–9.72)	0.109
Angiolymphatic invasion		
Absent vs present	5.95 (2.81–12.57)	<0.05*

*CI* confidence interval, *OR* odds ratio

*Statistically significant

**Table 5:** Multivariate analysis of histopathological predictors of lymph node metastases

Variables	OR (95 % CI)	*p* value
Tumor size		
≤2 vs >2 cm	2.22 (1.31–3.78)	0.003*
Differentiation		
I + I–II vs II + II–III + III	3.73 (1.54–9.06)	0.004*
Depth of invasion		
m1 to m3 vs sm1 to sm3	3.99 (1.53–10.40)	0.005*
Angiolymphatic invasion		
Absent vs present	4.64 (2.05–10.52)	<0.05*

*CI* confidence interval, *OR* odds ratio

*Statistically significant

## Discussion

SESC is defined as a tumor invading the mucosa and submucosa. It was difficult for patients with SESC to get early diagnosis and treatment in the past because of lack of any subjective symptoms. From the 1980s to the 2000s, due to the progress in flexible endoscopic procedure and biopsy [15], the incidence of SESC increased from 10 % to approximately 25 %. Recently, a number of studies reported that the prevalence of SESC ranged from to 16.9 to 34 % [3, 16–21].

Compared with advanced esophageal squamous cancer, the incidence of LNM in SESC is much lower. Since the lymph node status is the strongest prognosticator of survival of patients suffering from esophageal cancer [22–24], the SESC without LNM is potentially curable. Recent publications showed a lower incidence of LNM (0–5.6 %) in the m1 to m2 layer [18, 25, 26]. On the other hand, incidence of LNM in the sm2 to sm3 layer ranged from 18 to 67 % [26–28]. In our study, the incidence of LNM was 0.0 % of patients with m1 lesions, 2.7 % of patients with m2 lesions, 15.9 % of patients with sm2 lesions, and 34.3 % of patients with sm3 lesions. We also found that the number of involved lymph nodes was significantly different between the m1 to m2 groups and sm2 to sm3 groups. Furthermore, the involved lymph nodes were seen in more regions of sm2 to sm3 cancer cases than those of m1 to m2. Considering the low prevalence of LNM of m1 to m2, it is rational not to require surgery for a cure because the morbidity and mortality rates of SR often exceed such a low ratio of LNM. Thus, ER is gradually considered as a reliable treatment for m1 to m2 with curative intent [12, 18, 25, 29, 30]. Compared with traditional biopsy and conventional imaging techniques (i.e., endoscopic ultrasonography or CT), it even seems that ER can improve the tumor staging based on the assessment of the samples obtained. However, ER must be appropriately used with curative intent without compromise for distant metastasis or high risk of lymphatic spread.

On the other hand, due to the high ratio of LNM, SR is obviously applicable to patients with the tumor depth of sm2 to sm3. According to the number and the regions of LNM in sm2 to sm3, two-phase (Ivor Lewis) or even three-phase transthoracic esophagectomy is chosen for a more extensive lymphadenectomy. This extensive lymphadenectomy could decrease or even clear the residual positive node lymphaticus at the expense of mortality, morbidity, and poor quality of life after surgery. Tachibana and associates [23] reported that the postoperative hospital mortality rate was approximately 5 % and the morbidity rate was 40 %. Stephen and coauthors [31] reviewed 310 patients in 12 national cancer institutions and reported that actual mortality rate was approximately 4.2 % and the complications of care rate were 57 %. The morbidities, such as anastomotic leak, recurrent laryngeal nerve paralysis, and pulmonary complications, still deteriorate the prognosis after esophagectomy.

Furthermore, our study focused on the LNM in the depth of m3 to sm1 tumors. The incidence of LNM in m3 and sm1 was 6.3 and 18.2 %, respectively. Between m1 to m2 and sm2 to sm3, the nodal metastasis proportion of patients with m3 to sm1 lesions cannot be neglected. Due to the limitation of ER for lymph node biopsy, we tried to find out any predictors for LNM in this subset of patients to prevent them from the presence of tumor cells after ER.

Based on the results of our study, patients with positive lymph nodes showed a statistically larger tumor size, poorer differentiation, and deeper tumor invasion than those with negative lymph nodes. These pathological variables were confirmed to be associated with LNM on both univariate and multivariate logistic regression analyses. Additionally, the positive pathological characteristics, including the maximum tumor size more than 2 cm, poor tumor differentiation (II + II–III + III), and tumor with angiolymphatic invasion, were found to be the independent predictive factors for LNM. These findings corresponded well with some earlier reports [21, 26, 32].

It is still controversial which treatment is suitable for patients with m3 to sm1 tumor invasion. In this study, we found that the proportion of number and regions of lymph node involvement in SESC patients increased from m1 to sm3. Univariate analyses identified that the tumor invasion depth of submucosa (sm1 to sm3) is a risk factor for LNM (*p* < 0.001). And on multivariate analysis, compared with mucosal infiltration, the presence of submucosal infiltration was presented to be an independent prognostic factor for nodal metastases (*p* = 0.005). These data are in line with several studies reported before [18, 25, 30, 32]. Therefore, we confirmed that ER is not a suitable treatment for patients with sm1 lesions. We also found some differences between sm1, sm2, and sm3. Compared with sm2 to sm3 lesions, tumors of sm1 lesions presented lower incidence of metastases. And the number and regions of positive lymph nodes also increased from sm1 to sm2 to sm3. Thus, the extent of lymphadenectomy remains controversial. Furthermore, due to the relatively low incidence of LNM, we recommend ER as a curative treatment for patients with m3 carcinoma who have no angiolymphatic invasion, with well tumor differentiation (I + I–II), and tumor < 2 cm in size.

Last but not the least, it is very important whether a true assessment of the depth of m3 invasion can be obtained from ER accurately. In fact, the exact preoperative differentiation between m3 and sm1 lesions is difficult due to limitations of ER, such as the piecemeal resection, incomplete thickness of the submucosa damaged by injection and cutting, and sample shrinking due to electrocution damage or the muscularis mucosae wrinkle. Besides, a technically well-performed resection and an accurate description of the invasion relative to the deep and lateral resection margins both perform important roles in the final report. Considering these aforementioned situations, if there is any hesitation in the final report of m3 invasion, SR should be recommended as a remedial treatment.

## Conclusions

For patients with SESC, EUS T staging and chest MSCT are highly recommended as preoperative routines (PET-CT is optional) to evaluate any metastasis. When node metastasis to mediastinum can be ruled out, we propose that ER be considered as a reliable treatment for m1 to m2 lesions and SR be the standard and irreplaceable therapy for sm2 to sm3 lesions. However, due to the relatively high prevalence of lymph node for sm1 tumors, we must be aware of the fact that ER for even superficial submucosal tumors should be considered with caution. Therefore, we recommend that patients with sm1 tumors should undergo esophagectomy with radical lymphadenectomy as the only effective treatment. As we have to aim at decreasing the operative trauma, morbidity, and mortality without compromising the presence of potential tumor cells, the treatment strategy for m3 lesions still remains controversial. We recommend ER as the initial procedure for diagnosis. Then, a successful pathologic report is needed to support this treatment. This successful pathologic report includes not only the accurate description of the tumor’s invasion depth (only muscularis mucosa invasion) but also the absence of any risk predictors for LNM as well as the unambiguous description of the tumor’s deep and lateral resection margins (without any invasion of the resection margins). Otherwise, SR is recommended for those m3 lesions with any hesitation on the reports of tumor description by both gastroenterologists and pathologists. Last, SR is recommended for sm1 lesions confirmed by EUS and for those with positive mediastinum lymph nodes indicated by MSCT or PET-CT.

## Abbreviations

ER: Endoscopic resection
LNM: Lymph node metastasis
MSCT: Multi-slice computed tomography
SESC: Superficial esophageal squamous carcinoma
SR: Radical surgical resection
